# First Report of Familial Juvenile Hyperuricemic Nephropathy (FJHN) in Iran Caused By a Novel *De Novo* Mutation (E197X) in *UMOD*

**DOI:** 10.4172/1747-0862.1000218

**Published:** 2016-05-29

**Authors:** Tahereh Malakoutian, Atefeh Amouzegar, Farzaneh Vali, Mojgan Asgari, Babak Behnam

**Affiliations:** 1 Hasheminejad Kidney Center, Hospital Management Research Center, Iran University of Medical Sciences, Tehran, Iran; 2 Department of Medical Genetics and Molecular Biology, Faculty of Medicine, Iran University of Medical Sciences (IUMS), Tehran, Iran

**Keywords:** Juvenile hyperuricemic nephropathy, Mutation, Genetic screening, Kidney dysfunction

## Abstract

Uromodulin (*UMOD*) gene mutation causes autosomal dominant Uromodulin-Associated Kidney Disease (UAKD), which in turn leads to end-stage renal disease. This is the first case report of a family with UAKD caused by a novel *de novo* mutation (E197X) in the *UMOD* gene. This case is a 28-year-old man with severely reduced kidney function [1]. No similar case was reported in his family history. This report highlights and reminds the importance of genetic screening in young patients involving kidney dysfunction, as the UAKD and some other kidney genetic diseases may be late-onset.

## Introduction

Familial Juvenile Hyperuricemic Nephropathy (FJHN) is a rare autosomal dominant disease characterized by hyperuricemia, gout and chronic renal disease [1–4], that can affect both gender, equally, at young age. Hyperuricemia results from reduced fractional excretion of urate due to a defect in its transport in the proximal tubule [5]. Renal impairment usually appears during the teenage years and slowly progress to 30s and leads to end stage renal failure within 10 to 20 years [6]. Renal ultrasound appearances have been reported showing reduced renal size with abnormal echogenicity and occasionally renal cysts in some kindred [7–9].

Histologic examination demonstrates chronic interstitial nephritis. The most remarkable feature is thickening and splitting of tubular basement membrane [10].

FJHN is caused by mutations in uromodulin gene (*UMOD*) that encodes uromodulin protein as a specific urate transporter channel and has been mapped on chromosome 16p11-p13 (16p11.2) [2]. *UMOD* function is not completely clear, although it is the most observed protein in the urine of healthy people. It may protect against urinary tract infections and help control the secretion of water in urine.

Mutations of the gene encoding uromodulin (*UMOD*) have been reported in several studies [1]. Most mutations in the *UMOD* change a single amino acid and alter the structure of the protein resulting in no release from kidney cells. Abnormal uromodulin may also trigger apoptosis of kidney cells and cause progressive kidney disease.

Untill now about 50 families have been reported in literature. This is the first case report from Iran that is related to a young male with hyperuricemia, renal failure and positive family history of gout and end stage renal disease.

## *UMOD* Gene Analysis

Genomic DNA was extracted from peripheral leukocytes using standard method (Table 1). The coding region of the *UMOD* gene and its intron-exon boundaries were amplified via PCR. The primers are listed (Table 2), and PCR conditions are available upon request. Single-strand sequencing was performed utilizing standard methods and gene specific primers via ABI 3730 (Applied Biosystems, Macrogen, South Korea). Sequences of all amplicons were compared with the published template (accession no. NM_003361.3) using Mutation Surveyor (version 3.20; Soft Genetics, State College, PA). Any changes in the sequence were checked against published polymorphisms and mutations and for conservation across species.

## Case Report

A 28 years old male, the first kid/sib of healthy parents with consanguineous marriage admitted to Hasheminejad hospital with hypertension, lower extremities edema, and high serum creatinine level. Past medical history was remarkable for hyperuricemia and gout. On admission the blood pressure was 160/90 and pitting edema of both lower limbs was found, other organs were otherwise normal. On sonographic examination right kidney was 114 mm and contained a thick wall cyst measured 23 × 24 mm and left kidney was 90 mm, with increased cortical echogenicity in the cortex of both kidneys. Doppler sonographic findings were RI=0.65 in right and 0.7 in left kidney.

**Figure 1:**
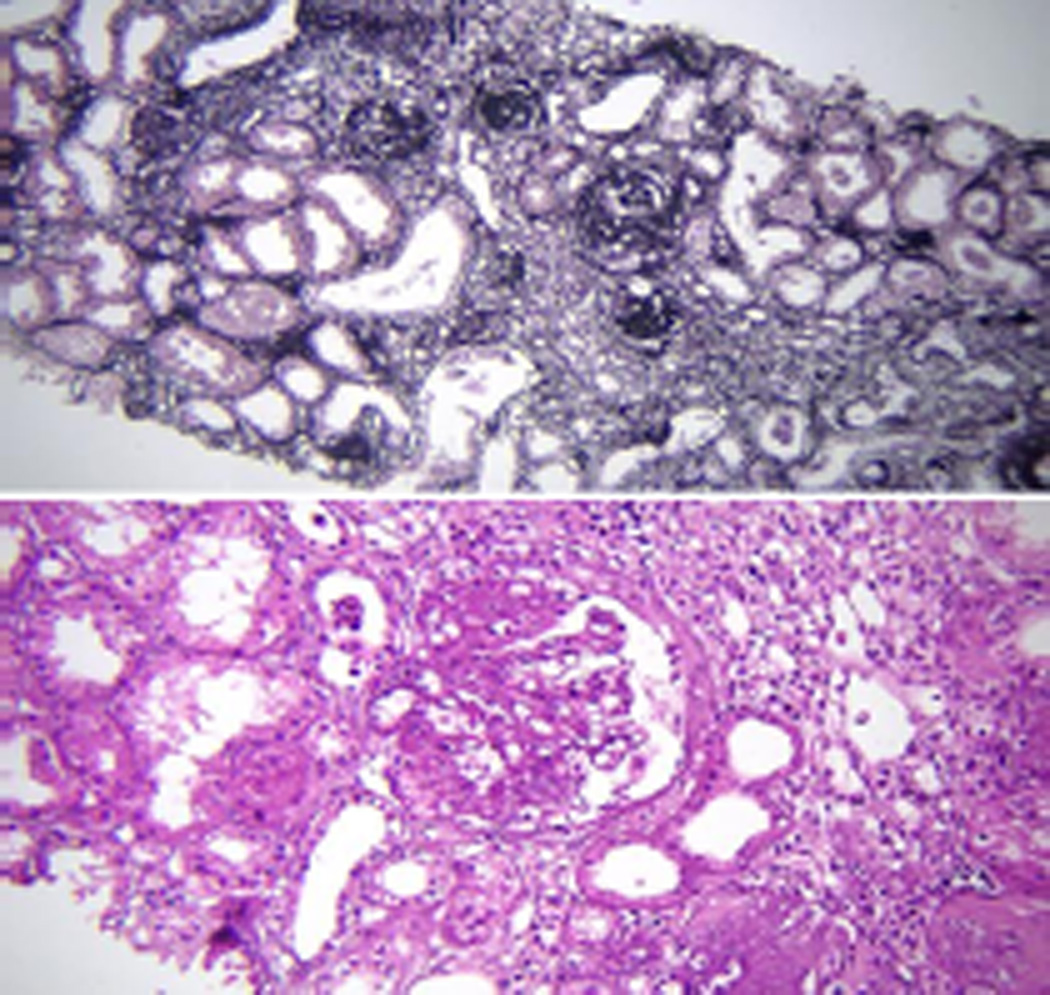
Top panel: Globally sclerosed glomeruli associated with atrophic tubules and chronic inflammatory cells infiltration of interstitium (×10 silver stain). Bottom panel: Segmentally large glomerulus and chronically inflamed interstitium associated with atrophic tubules (×20 H&E).

In the family history, his father and mother were cousins, his elder brother was diagnosed with gout and renal failure at age 33, and other positive findings were hyperuricemia and renal stone in his two cousins. Laboratory examination was as follow:

Due to renal failure and normal kidney size, a renal biopsy was done, which revealed advanced glomerulosclerosis in focal and segmental pattern, severe tubular atrophy and proportional fibrosis mostly secondary and mild to moderate arterionephrosclerosis (Figure 1).

**Figure 2:**
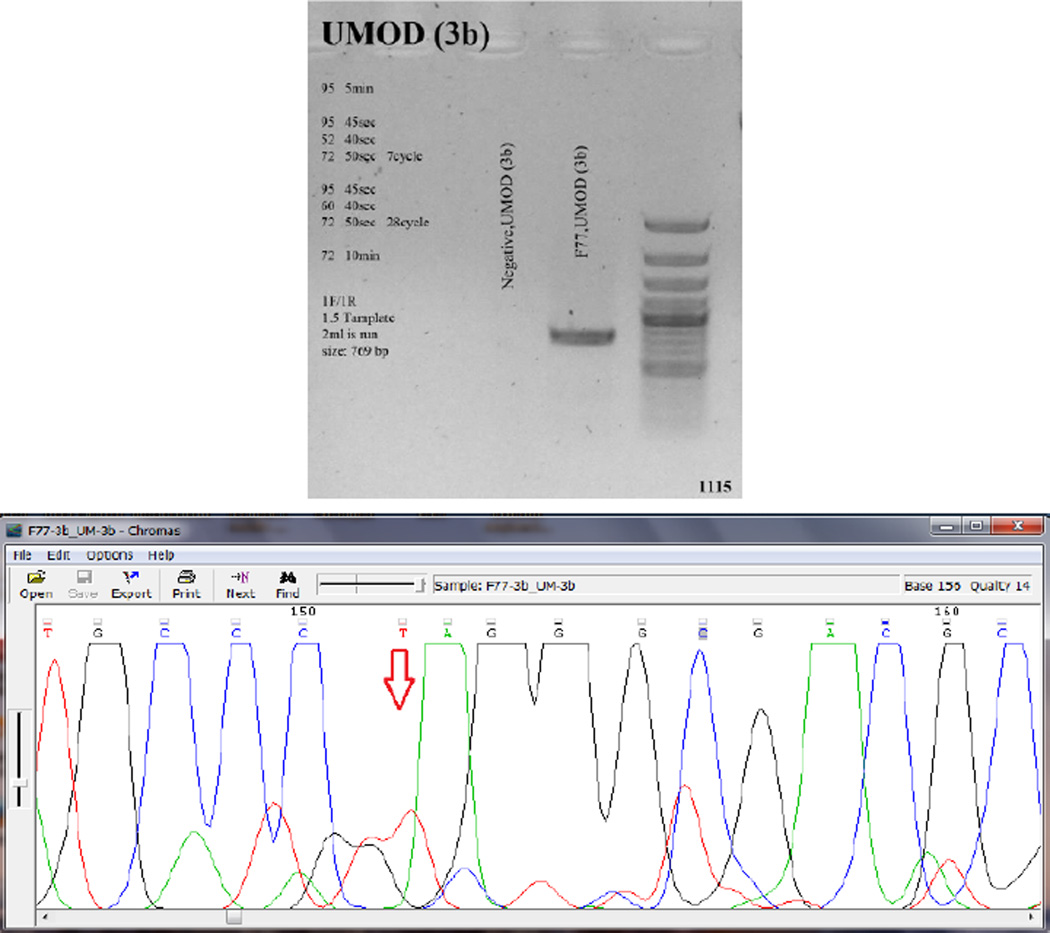
Amplification and Sequence Analysis of *UMOD* exon 3. Top: Genomic PCR amplifies the exon 3 using the gene-specific pair of primers (3b). Bottom: Corresponding chromatogram (Chromas software version 2.4.1) for the region containing alterations. Red arrow shows the substituted nucleotide.

We proposed Familial juvenile hyperuricemic nephropathy as the most possible diagnosis for this patient with respect to clinical manifestations, paraclinical findings, and family history, which led us to perform genetic study to confirm the diagnosis. Sequencing analysis showed that the patient is homozygous for a novel nonsense mutation, c.589G>T; p.E197X, in exon 3 of the *UMOD* gene (Figure 2). This confirms and interprets his involvement in familial juvenile hyperuricemic nephropathy.

## Discussion

Familial juvenile hyperuricemic nephropathy (FJHN) is a rare autosomal-dominant disorder characterized by hyperuricemia and decreased urinary excretion of urate, chronic interstitial nephritis, and result in progressive renal failure [11,12]. The basic mechanism of the correlation between early hyperuricemia and subsequent progressive renal involvement is unclear. FJHN and autosomal-dominant Medullary cystic kidney disease (MCKD) overlap in some clinical features and manifestations. MCDK is also a rare disease with progressive chronic interstitial nephritis during adulthood, associated with an inconstant observation of corticomedullary cysts [13–16].

The purine metabolism end product -urate- [17], is freely filtered by the glomerulus and mainly reabsorbed, as only 10% of its primary filtration exerts in urine [18]. Despite well recognition of the urate transport mechanisms in the proximal tubule (PT), its permeability in the distal segments of the nephron has not been described [19].

Uromodulin (or Tamm-Horsfall) protein as the major component of urinary casts and the most abundant urine protein component is specifically synthesized in the thick ascending limb of the Henle loop. As a pro-inflammatory protein, it may activate neutrophils, and stimulate monocyte proliferation for cytokines and gelatinases releases [20]. A significant reduced urinary level of *UMOD* in the patients with FJHN has been documented by several independent survies [21,22].

*UMOD* mutation may cause either medullary cystic kidney disease type 2 (MIM 603860) or familial juvenile hyperuricemic nephropathy (MIM 162000). Therefore, as uromodulin-associated kidney disease (UAKD) [23], both are autosomal dominant tubulointerstitial kidney disorders.

To date, 51 *UMOD* mutations have been reported which all of them (except three in-frame deletions) are nonsense variations. In more than 50% (28/51) of these mutations, one of the conserved cysteine residues is affected. Meanwhile most of the *UMOD* mutations cause protein misfolding, via affecting the disulfide bond or destabilizing the structure of EGF-like domains. *UMOD* mutations are mainly clustered (94%) in the N-terminal half of the protein encoded by exons 3 and 4. Only three reported mutations occurred in exons 5 and 7 affecting residues within the ZP domain [24–26].

A decrease in renal failure progression via lowering serum urate is still controversy, due to a start with a xanthine oxidase inhibitor early in the course of the disease in the studies reporting benefit [27].

This study reports the first case of UAKD in Iran. Molecular genetic analysis revealed a heterozygote substitution, c.589G>T; p.E197X. Glutamic acid 197 resides in a highly conserved domain of the protein that is preserved among human, chimpanzee, mouse, rat, pig, bovine and sheep.

To date, more than 14 mutations have been described to cause FJHN, mostly in coding regions (Table 3). There does not appear to be a “hot spot”, missense and nonsense mutations are scattered in coding regions and splice sites. The majority of alterations that lead to UAKD are missense mutations resulting in amino acid change and consequently the proper function of protein. Some reports also described mutations in non-coding regions, which mostly affect mRNA splicing (such as IVS1+2T>C and g.IVS1+2_3insT) [28,29]. Recently, in a family that showed C135G mutation in *UMOD*, Kuma and colleagues discovered a moderate kidney filtration abnormality [30].

In most of the FJHN patients, a *UMOD* mutation has been detected in an autosomal dominant manner. By the other word, various *UMOD* heterozygous missense mutations have been revealed in the families with FJHN.

## Figures and Tables

**Table 1:** Laboratory tests of the patient involved in FJHN.

Lab test	Value
FBS	101 mg/dl
BUN	106 mg/dl
Cr	8.5 mg/dl
Uric acid	13.4 mg/dl
Calcium	9.7 mg/dl
Phosphorus	8.2

**Table 2:** List of UDO-specific primers.

UMOD-1F	CTCTTGCTGTTAGAAGGTGCGA	574 bp
UMOD-1R	GCAAGTTGTTCATTGTTGTAACCC	
UMOD -2F	GGTGAGTCCAGAACACTATTCCA	721 bp
UMOD -2R	ATTCAGCATCTCAGGTCCTACTG	
UMOD -3Fa	CTTGACATCATCAGAGGAGTTTTG	530 bp
UMOD -3Ra	ACTCACAGTGCCATCCATCC	
UMOD -3Fb	CTGCACAGACGTGGATGAGT	769 bp
UMOD -3Rb	CTCACCAGTGATGTTGAAGTCC	
UMOD -4F	CGGCTACTACGTCTACAACCTGAC	436 bp
UMOD -4R	TGCCGGCTTTAATGGGTATTAG	
UMOD -5F	GGTATATAACCCACATTTAGGGGAAC	761 bp
UMOD -5R	CCTACTATGGCCCTACTTTTCCC	
UMOD -6F	CAGACATGAGACCAGCAGATTTAG	540 bp
UMOD -6R	AGCCAGGCTTAATAACTCACTCAA	
UMOD -7F	CAAGTTGACCTGCGTGTACTTATT	853 bp
UMOD -7R	AATGCTAAGGTTCAGCTAAAGGG	
UMOD -8F	CTATGGCATGCTACACACAGTTC	587 bp
UMOD -8R	ATCCCACCTGATTTCCCCT	
UMOD -9F	GGTATTACTGGTTCCCTTTCCTCA	558 bp
UMOD -9R	CCGTGTCCTGTGTTACATTCATC	
UMOD -10F	AGGGTTGGGACCTTTCTCC	412 bp
UMOD -10R	GAGAGATGCATGATCTCAGTAGGAC	
UMOD -11F	TCGGTCCACCTTTTTCAGG	442 bp
UMOD -11R	CAGGTACACCGTCACAAGTCC	

**Table 3:** List of known mutations demonstrated by ClinVar. (http://www.ncbi.nlm.nih.gov/clinvar/?term=umod%5Bgene%5D)

	Variation/Location	Clinical significance (Last reviewed)
1	27-BP DEL, NT1966	Pathogenic (Dec 1, 2002)
2	c.943T>C (p.Cys315Arg)	Pathogenic (Dec 15, 2003)
3	c.898T>G (p.Cys300Gly)	Pathogenic (Mar 1, 2003)
4	c.817G>T (p.Val273Phe)	Pathogenic (Sep 1, 2006)
5	c.764G>A (p.Cys255Tyr)	Pathogenic (Mar 1, 2003)
6	c.743G>C (p.Cys248Ser)	Pathogenic (May 15, 2010)
7	c.649T>G (p.Cys217Gly)	Pathogenic (Jan 6, 2012)
8	c.649T>C (p.Cys217Arg)	Pathogenic (Dec 17, 2004)
9	c.443G>A (p.Cys148Tyr)	Pathogenic (Dec 1, 2002)
10	c.383A>G (p.Asn128Ser)	Pathogenic (Mar 1, 2003)
11	c.376T>C (p.Cys126Arg)	Pathogenic (Jan 6, 2012)
12	c.307G>T (p.Gly103Cys)	Pathogenic (Dec 1, 2002)
13	c.230G>A (p.Cys77Tyr)	Pathogenic (Mar 1, 2003)
14	c.-1746T>C	association (Jul 1, 2015)
